# Quantifying the Burden and Trends of Isoniazid Resistant Tuberculosis, 1994–2009

**DOI:** 10.1371/journal.pone.0022927

**Published:** 2011-07-29

**Authors:** Helen E. Jenkins, Matteo Zignol, Ted Cohen

**Affiliations:** 1 Brigham and Women's Hospital, Boston, Massachusetts, United States of America; 2 Harvard Medical School, Boston, Massachusetts, United States of America; 3 Stop TB Department, World Health Organization, Geneva, Switzerland; 4 Department of Epidemiology, Harvard School of Public Health, Boston, Massachusetts, United States of America; San Francisco General Hospital, University of California San Francisco, United States of America

## Abstract

**Background:**

Quantifying isoniazid resistant (INH-R) tuberculosis (TB) is important because isoniazid resistance reduces the probability of treatment success, may facilitate the spread of multidrug resistance, and may reduce the effectiveness of isoniazid preventive therapy (IPT).

**Methodology/Principal Findings:**

We used data reported to the World Health Organization between 1994–2009 to estimate the INH-R burden among new and retreatment TB cases. We assessed geographical and temporal variation in INH-R and reported levels in high HIV prevalence countries (≥2%) to understand implications for IPT. 131 settings reported INH-R data since 1994. A single global estimate of the percentage of incident TB cases with INH-R was deemed inappropriate due to particularly high levels in the Eastern European region where 44.9% (95% CI: 34.0%, 55.8%) of incident TB cases had INH-R. In all other regions combined, 13.9% (95% CI: 12.6%, 15.2%) of incident cases had INH-R with the lowest regional levels seen in West/Central Europe and Africa. Where trend data existed, we found examples of rising and falling burdens of INH-R. 40% of high HIV prevalence countries reported national data on INH-R and 7.3% (95% CI: 5.5%, 9.1%) of cases in these settings had INH-R.

**Conclusions/Significance:**

Outside the Eastern European region, one in seven incident TB cases has INH-R, while this rises to nearly half within Eastern Europe. Many countries cannot assess trends in INH-R and the scarcity of data from high HIV prevalence areas limits insight into the implications for IPT. Further research is required to understand reasons for the observed time trends and to determine the effects of INH-R for control of TB.

## Introduction

In 2009, the World Health Organization (WHO) estimated that there were 9.4 million incident cases of tuberculosis (TB) and 1.7 million deaths due to TB [1]. While the incidence of TB is decreasing in five of the six WHO regions [1], the emergence of multi-drug resistant (MDR) TB (TB which is resistant to at least isoniazid and rifampin) [2] threatens to derail advances made against TB. MDRTB can take up to two years or more to treat with drugs that are less potent, more toxic and much more expensive [2]. The most recent WHO summary of the global burden of drug resistant TB was released in 2010 and reported the highest levels recorded for MDRTB, with an estimated 3.6% of incident TB resistant to the two most important first line antibiotics [2].

Although combined resistance to essential first-line antibiotics (i.e. MDR) poses the greatest risk to patients, resistance to isoniazid alone is also important. Isoniazid is the cornerstone of any first-line treatment. Compared with other TB drugs, it has the most potent early bactericidal activity [3], [4] and rarely causes adverse events [5]. Loss of effectiveness of this drug compromises both the preventive therapy and treatment of disease. Recent systematic reviews and meta-analyses confirmed that isonaizid monoresistance is associated with reduced probability of successful treatment outcomes for individuals receiving standard treatment and an increased risk of acquiring additional drug resistance [6], [7]. In addition, individuals infected with isoniazid resistant strains of *M. tuberculosis* are unlikely to benefit from Isoniazid Preventive Therapy (IPT), which, in the absence of resistance, is effective in reducing the risk of progression to disease after infection [8]. Given the growing role of IPT in addressing the challenge of HIV-associated TB [9], measuring the burden of isoniazid resistant (INH-R) TB in populations with high prevalence of HIV is important for understanding if resistance will compromise the expected benefits of this intervention.

In this paper, we use all data from population-representative surveys and surveillance on INH-R reported to the WHO between 1994 and 2009. We estimate the burden of INH-R regionally and nationally and examine trends where data are available.

## Methods

### Data collection

In 1994, the International Union Against Tuberculosis and Lung Diseases and the Stop TB Department at the WHO formed a Global Project on Anti-Tuberculosis Drug Resistance in order to estimate the worldwide burden of drug resistant TB. Through this initiative, national and sub-national data from surveillance activity (in countries where continuous drug sensitivity testing of all incident TB cases is performed) and periodic population representative surveys (in countries where drug sensitivity tests are not routinely performed) have been reported to the WHO in standard formats permitting the generation of regional and global estimates of the burden and trends of resistant disease. The details of these surveillance and survey activities have been provided elsewhere [2], [10]. Data in this analysis include all reports of surveillance and survey activities made to the Global Project relating to the period 1994–2009. All of the data used here have been published previously [2], [11], [12], [13], [14] (although never previously analysed to address these questions) and have been cleared for publication by individual countries.

### Statistical analysis

We examined three categories of isoniazid resistance: resistance to isoniazid (in the absence or presence of any other drug resistance), resistance to isoniazid (in the absence or presence of any other drug resistance *with the exception of rifampin*), and multidrug resistance (isoniazid resistance in the presence of rifampin resistance). To assess availability of data, we categorized countries according to whether, from 1994 to 2009, they reported data once, twice or three or more times and whether or not they reported data at a national or sub-national level. We also examined whether countries had reported any data since 2000 to assess the representativeness of existing data for current conditions.

We estimated the mean percentages of individuals with these three types of resistance among three groups: new TB cases (those that have received less than 1 month of TB treatment at any point in the past), retreatment TB cases (those that have at least 1 month of TB treatment at any point in the past), and all TB cases (i.e. combined category of new and retreatment cases). We estimated the percentage with each of the three types of resistance among these three groups using the most recent information from all countries that reported data between 1994 and 2009 and weighted by population size. Estimates were based on countrywide data where available and from sub-national surveys where national estimates were not reported. We then used these national/subnational estimates to produce estimates by WHO region and for the world. Since all estimates were weighted by population size, we excluded sub-national surveys conducted in areas where population size was not reported from these aggregated estimates. Results were based on reported data only; no estimates were made for countries that did not report data.

We also produced mean estimates for countries with high prevalence of HIV among adults, which we defined as those countries with an estimated adult seroprevalence of 2% or higher in 2009 [15].

For most analyses in this report, we report the *percentage* with each type of resistance; however, to better characterize time trends in resistance, we also provide estimated trends in the *number* of TB cases with resistance. Each measure provides useful information and considering both together facilitates accurate interpretation of changes over time. For example, if the incidences of both drug-resistant and drug-sensitive TB are falling, but resistant disease is declining at a slower pace, then the percentage of incident cases with resistance will increase over time despite an otherwise promising reduction in burden [16].

We estimated the number of new TB cases with INH-R by multiplying our estimated percentage of new TB cases with INH-R by the number of new notified TB cases in that setting. Per capita rates were calculated based on reported population size. Because the estimated numbers of new cases are considered more reliable, we did not estimate the incidence of drug-resistance among cases presenting for retreatment.

We estimated time trends for each setting that reported data at least three times between 1994 and 2009. We used a weighted least squares regression and modelled the percentage of new TB cases with INH-R using year as the explanatory variable and weighted by the number of cases tested in each year. We looked for a significant linear effect of year to identify increasing or decreasing trends (using a p-value of 0.1 to conservatively ensure that any changes were detected). Note that all estimated changes in the percentage of cases with resistance were absolute changes, i.e. from a starting point of 5%, a 5% increase represents an increase to 10%. We performed similar analyses for the number of new TB cases with INH-R per 100,000 population, weighting by the population each year in this case. To further explore trends in INH-R, we subdivided the numbers of new TB cases with INH-R per 100,000 population into those with INH-R but not rifampin resistance and those with MDR. In this analysis, we only examined numbers and not percentages since changes in numbers have clearer relevance for the burden of disease over time. Note that areas with non-significant time trends may still report substantial differences from one time point to the next. However, our aim here was to identify areas where there is evidence that INH-R levels are changing consistently over time.

SAS version 9.2 and R version 2.11.1 were used for all analyses.

## Results

### Data availability

**Figure 1:**
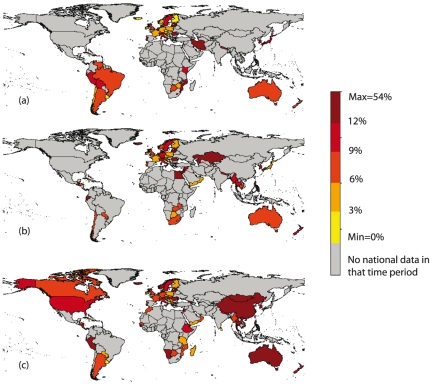
Percentage of incident TB cases with isoniazid resistance. World map showing the percentage of incident TB cases with isoniazid resistance (INH-R) from the most recent survey in each setting in three time periods: (a) 1994-1999, (b) 2000-2004, (c) 2005-2009. Grey areas indicate those that did not report national data in the time period in question.

Since 1994, 106 countries/territories have provided national data on INH-R among all incident TB cases (Figure 1). Including countries reporting only sub-national data, 131 different settings provided data on all incident cases, of which 39% provided sufficient data to estimate time trends (3 or more data points). Data provided on INH-R among all TB cases cover 56% of the world's population.

### Isoniazid resistance - global estimates and geographic variation

**Figure 2:**
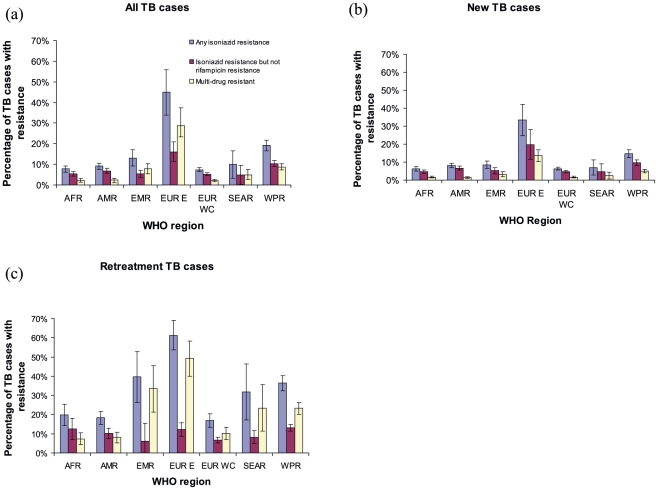
Percentage of all, new and retreatment TB cases with isoniazid resistance by WHO region. Percentage of (a) all TB cases, (b) new TB cases and (c) retreatment TB cases with any resistance to isoniazid, resistance to isoniazid but not rifampin and multi-drug resistance by WHO region (AFR =  African region, AMR =  region for the Americas, EMR =  Eastern Mediterranean region, EUR E =  Eastern European region, EUR WC =  Western and Central European regions, SEAR =  South-East Asian region, WPR =  Western Pacific region). Countrywide data only from the most recent survey available 1994-2009. Blue bars represent any isoniazid resistance, red bars represent isoniazid resistance without rifampin resistance and white bars represent multi-drug resistance.

The estimated percentage of incident TB cases with INH-R was substantially higher in the Eastern European region than any other WHO region (Figure 2) at 44.9% (95% CI: 34.0%, 55.8%). Of new cases, 33.5% (95% CI: 24.8%, 42.2%) had INH-R and 61.4% (95% CI: 53.6%, 69.2%) of retreatment cases had INH-R. A single global estimate, therefore, seemed inappropriate and we focus instead on the Eastern European region and all other regions separately. All other regions combined reported an estimated 13.9% (95% CI: 12.6%, 15.2%) of incident cases with INH-R (10.7% (95% CI: 9.8%, 11.7%) among new TB cases and 29.0% (95% CI: 26.4%, 31.5%) among retreatment cases). The Western and Central European countries had the lowest percentages of INH-R among all incident cases at 6.4% and 17.0% among new and retreatment cases respectively closely followed by the African region at 6.3% and 20.0% (Figure 2).

The highest percentages of INH-R among new TB cases (40-43%) were found in Kazakhstan, Baku City in Azerbaijan and Arkhangelsk in the Russian Federation. Outside the Eastern European regions, China, Vietnam, the Dominican Republic and parts of India reported between 15% and 20% of new TB cases with INH-R. Among retreatment cases, the highest percentages with INH-R were in Baku City in Azerbaijan (80%) and some Russian Oblasts (>70%). Outside the Eastern European region, the highest percentages were in Lebanon (75%), Sierra Leone (62%) and parts of China (45–55%).

### Isoniazid resistance – time trends

**Figure 3:**
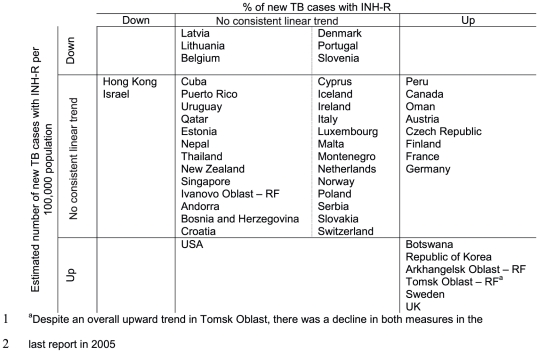
Linear trends in isoniazid resistance (INH-R) among new TB cases. We estimated trends in the percentage of new TB cases with INH-R and the estimated number of new TB cases with INH-R per 100,000 population. Settings are grouped by any linear trend (p<0.1) found (“down” or “up”) or “no consistent linear trend” if no linear trend was found. RF = Russian Federation.

**Figure 4:**
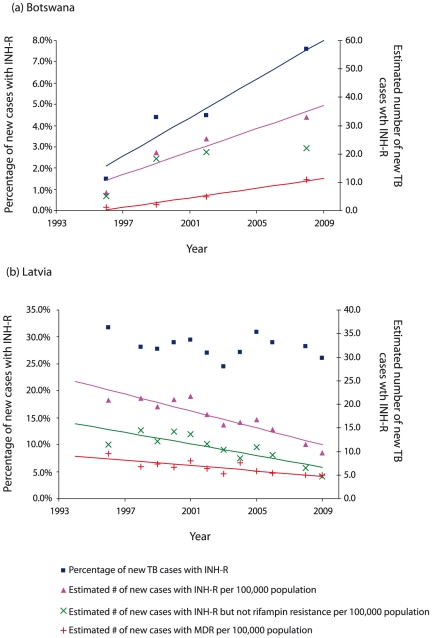
Percentage and number of new TB cases with isoniazid-resistant TB in (a) Botswana and (b) Latvia. Markers indicate reported data. The numbers with INH-R per 100,000 population (pink triangles) are also subdivided into those with MDR (red ‘+’ symbols) and those with INH-R without rifampin resistance (green ‘X’ symbols). Data points for the percentage of TB cases with INH-R are marked by blue squares. Lines indicate fitted linear trends and are *only* displayed where a significant linear trend was found (p<0.1). The colour of the line matches the colour of the data points to which the linear trend is fitted. Note that scales are different between (a) and (b) and that there are two y-axes, one for the percentage and one for the number of TB cases with INH-R per 100,000 population.

Due to the scarcity of time series data, it was not possible to assess global or regional trends in INH-R TB. However, time series analysis was possible for 51 and 46 settings that reported data on INH-R in new and retreatment TB cases respectively at least three times between 1994 and 2009. Here we highlight results for settings with particularly strong trends and/or substantial estimated annual changes (Figure 3). For further illustration in Figure 4(, we show more detailed time trends for a) Botswana, a country with high HIV prevalence that is currently increasing implementation of IPT and (b) Latvia, a country with a large burden of drug resistant TB that has made substantial commitments to the control of drug resistance.

#### Increasing percentages and numbers of new TB cases with INH-R

Both the estimated percentage and number of new TB cases with INH-R per 100,000 increased significantly in the Republic of Korea and Botswana (estimated annual increase of 2 cases per 100,000 in Botswana, Figure 4a). In the Russian Federation, the percentage of new TB cases with INH-R in Arkhangelsk Oblast increased from 2002–05 with an estimated absolute annual increase of 5.0% (i.e. 5 percentage points each year).

#### Increasing percentages but no consistent linear trend in numbers of new TB cases with INH-R

The estimated percentage of new cases with INH-R increased in Peru, Canada, Finland and France while the estimated number of new TB cases with INH-R per 100,000 showed no consistent linear trend. In Oman, the percentage of new cases with INH-R has increased by an estimated 1.25% annually since 2004 (i.e. an absolute increase of 1.25 percentage points annually).

#### Decreasing trends in percentages and/or numbers of new TB cases with INH-R

The estimated percentage of new cases with INH-R decreased in Israel (1.0% absolute annual decrease) and in Hong Kong although there has been a slight increase observed between 2007 and 2009. There have been steady declines in the estimated number of new TB cases with INH-R per 100,000 in Latvia and Lithuania (estimated annual decreases of 0.9 and 0.4 cases per 100,000 respectively, Figure 4b) and also in Belgium and Portugal.

### Isoniazid resistance – with and without rifampin resistance

Subdividing the number of new TB cases with INH-R into those with MDR and those with INH-R without rifampin resistance aids understanding of underlying mechanisms for observed trends in INH-R TB.

**Figure 5:**
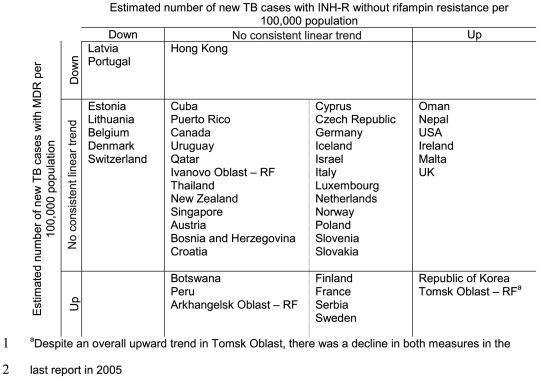
Trends in the number of new TB cases per 100,000 population with INH-R. Settings were included that provided at least 3 data points from 1994 to 2009. Trends were estimated for two types of INH-R: INH-R with rifampin resistance (multi-drug resistance (MDR)) and INH-R without rifampin resistance. Settings are grouped by any linear trend (p<0.1) found (“down” or “up”) or “no consistent linear trend” if no linear trend was found. RF = Russian Federation.

In Figure 5, we show those settings that have reported changes in the estimated number of new TB cases with MDR and those with INHR without rifampin resistance. In some settings, changes in INH-R appear to be driven primarily by changes in MDR and in others by changes in INH-R without rifampin resistance.

### Isoniazid resistance in high HIV prevalence settings

Of the 29 countries reporting HIV prevalence of 2% or more[15], 14 (48%) have reported data on INH-R in new and retreatment cases. Two provided data only at a sub-national level and 3 of those providing national data did so only before 2000.

Among those countries reporting HIV prevalence of 2% or more and reporting nationwide data, 7.3% (95% CI: 5.5%, 9.1%) of all incident TB cases had INH-R (6.5% of new cases and 17.6% of retreatment cases). Botswana was the only setting in this group that reported time series data (3 or more data points); it has seen increasing trends in INH-R in new cases (Figure 4a).

## Discussion

There is considerable geographic heterogeneity in the percentage of TB cases with resistance to isoniazid. In the Eastern European WHO region, nearly half of incident TB cases have INH-R; one in three new cases have INH-R (indicating the extent of transmission of this type of resistance) and 60% of retreatment cases. In the rest of the world, around one in seven incident TB cases have resistance to this important first line drug.

Data on INH-R in TB cases have been provided for 131 settings since 1994 representing just over half of the world's population. However, many of these areas have provided data at only a single time point, sub-nationally or more than a decade ago. Only around 40% of these settings have provided data at 3 or more time points between 1994 and 2009 limiting our ability to assess trends. Surveillance for INH-R is important because resistance to isoniazid increases the probability of acquiring resistance to other drugs including rifampin and thereby MDR-TB [6], [7], [17]. The presence of INH-R also compromises the likelihood of having a successful outcome with treatment [6], [7], [17]. Testing and reporting is currently not sufficiently frequent to monitor the levels or trends in isoniazid resistance for many countries.

Monitoring of INH-R is especially important in places where IPT may be initiated or its coverage extended. In countries with HIV prevalence of 2% or more, the percentage of incident cases with INH-R is 7.3%; this is lower than the overall average for countries reporting data to the WHO (even excluding Eastern Europe), but does suggest that a substantial number of individuals receiving IPT may already be resistant to this drug. We also note that there is limited availability of TB resistance data from high HIV prevalence countries [18]; only one-third of these countries with adult HIV seroprevalence of at least 2% have reported national data since 2000. Botswana, the one country where time trend analysis is possible, has had an apparent increase in both the percentage and number of new TB cases with INH-R; this is appears to be mostly due to rising MDR and is concerning because this is the African setting in which IPT roll-out is being adopted most rapidly [19], [20], [21]. The evidence is compelling that IPT could reduce incidence of active disease substantially in these high HIV prevalence areas [8] but the presence of INH-R may reduce these expected benefits. Continued monitoring of INH-R in areas in which IPT is being utilized will be essential for ensuring the effectiveness of preventive therapy.

Given that INH-R increases the likelihood of acquiring additional resistance during standard TB treatment, monitoring the burden of INH-R (and INH-R without rifampin resistance in particular) may provide important early warnings for areas vulnerable to increases in MDRTB. Figure 5- shows the settings in which numbers of TB cases with INHR without rifampin resistance are increasing. During the study period, the number of new TB cases with this type of resistance increased in a number of geographically disparate settings such as the Tomsk Oblast in the Russian Federation, the Republic of Korea, Oman and the USA. Such trends are concerning not only for the impact on treatment outcomes but also the potential subsequent implications for MDRTB. It should also be noted that, while not the main focus of this paper, Figure 5 highlights a number of countries with increasing numbers of MDRTB cases per capita.

Within Eastern Europe - the region with the highest percentage of incident cases with INH-R - the current situation is worrisome, but the outlook is improving in some locations. The number of new TB cases with INH-R among new cases is declining, for example, in Lithuania and Latvia (Figure 4b). In the latter, reductions in the numbers of incident TB cases with drug resistance have been linked to several factors including improved case detection, individualized treatment and case management strategies and better quality drug supply and distribution minimising interruptions to treatment [22].

In settings where the current burden of INH-resistance is currently low, upward trends should be viewed with concern. Such trends were apparent in countries such as Botswana, Republic of Korea and Peru. There are also parts of Western Europe, such as France, where despite decreases in TB notifications [23], the percentage and/or number of new TB cases with INH-R is increasing.

There are also some clear success stories such as Lithuania and Latvia, as described above and some Western European countries that have seen a decline in numbers of cases with INH-R without rifampin resistance such as Belgium, Switzerland and Denmark. In other locations, the percentage of new cases with INH-R is on a downward trend such as in Hong Kong where high case detection rates and a low proportion of cases requiring retreatment are indicative of a well functioning TB control programme [24]. In Portugal, the number of new TB cases with INH-R has declined and this may at least partially reflect the effect of efforts to improve case detection and treatment completion among individuals struggling with drug addiction, a risk group with high rates of treatment abandonment [25].

These data raise further questions: at the population level, does increasing incidence of INH-R precede increasing incidence of MDR? Does INH-R increase – or decrease – more rapidly over time than MDR? More detailed resistance data, especially those that include repeated sampling or surveillance in high TB incidence countries will help address some of these important questions.

Molecular methods for drug sensitivity testing of TB strains will markedly reduce the turn around time for testing. However, since there is a far greater understanding of the mutations that are associated with rifampin resistance than isoniazid resistance [26], sensitivity for detecting INH-R with these methods is currently far lower than for rifampin resistance [27]. In fact, since resistance to rifampin in most settings is usually accompanied by INH-R, some tests that are being used to screen for MDRTB only test for rifampin resistance [28], [29]. Unless additional mutations associated with isoniazid resistance are identified to increase the sensitivity of these molecular tests, there is a possibility that surveillance and detection of INH-R will decrease rather than increase in the future.

The regional averages that we have provided are from countries that have reported data and therefore may not be representative of areas in which data were not available (Figure 2). Through this analysis of existing population representative data on INH-R TB, we have identified areas of concern as well as encouraging examples of settings with declining burdens of isoniazid resistance. A key additional finding is the absence of recent drug resistance surveillance data from many settings. Since the ability to respond most effectively to the threat of drug-resistant TB requires an understanding of the magnitude of this problem, we encourage continued and increased financing of these essential monitoring activities.
